# Mechanism of Histone H3K4me3 Recognition by the Plant Homeodomain of Inhibitor of Growth 3*[Fn FN2]

**DOI:** 10.1074/jbc.M115.690651

**Published:** 2016-06-08

**Authors:** Sophia Kim, Senthil Natesan, Gabriel Cornilescu, Samuel Carlson, Marco Tonelli, Urszula L. McClurg, Olivier Binda, Craig N. Robson, John L. Markley, Stefan Balaz, Karen C. Glass

**Affiliations:** From the ‡ Department of Pharmaceutical Sciences, Albany College of Pharmacy and Health Sciences, Colchester, Vermont 05446,; the § National Magnetic Resonance Facility at Madison and Department of Biochemistry, University of Wisconsin-Madison, Madison, Wisconsin 53706, and; the ¶ Newcastle Cancer Centre at the Northern Institute for Cancer Research, Newcastle University, Newcastle Upon Tyne NE2 4HH, United Kingdom

**Keywords:** epigenetics, histone methylation, isothermal titration calorimetry (ITC), nuclear magnetic resonance (NMR), PHD finger, Inhibitor of Growth 3, histone acetyltransferase, plant homeodomain

## Abstract

Aberrant access to genetic information disrupts cellular homeostasis and can lead to cancer development. One molecular mechanism that regulates access to genetic information includes recognition of histone modifications, which is carried out by protein modules that interact with chromatin and serve as landing pads for enzymatic activities that regulate gene expression. The ING3 tumor suppressor protein contains a plant homeodomain (PHD) that reads the epigenetic code via recognition of histone H3 tri-methylated at lysine 4 (H3K4me3), and this domain is lost or mutated in various human cancers. However, the molecular mechanisms targeting ING3 to histones and the role of this interaction in the cell remain elusive. Thus, we employed biochemical and structural biology approaches to investigate the interaction of the ING3 PHD finger (ING3_PHD_) with the active transcription mark H3K4me3. Our results demonstrate that association of the ING3_PHD_ with H3K4me3 is in the sub-micromolar range (*K_D_* ranging between 0.63 and 0.93 μm) and is about 200-fold stronger than with the unmodified histone H3. NMR and computational studies revealed an aromatic cage composed of Tyr-362, Ser-369, and Trp-385 that accommodate the tri-methylated side chain of H3K4. Mutational analysis confirmed the critical importance of Tyr-362 and Trp-385 in mediating the ING3_PHD_-H3K4me3 interaction. Finally, the biological relevance of ING3_PHD_-H3K4me3 binding was demonstrated by the failure of ING3_PHD_ mutant proteins to enhance ING3-mediated DNA damage-dependent cell death. Together, our results reveal the molecular mechanism of H3K4me3 selection by the ING3_PHD_ and suggest that this interaction is important for mediating ING3 tumor suppressive activities.

## Introduction

Although the human genome was sequenced over a decade ago and its structure has been investigated for nearly half a century, the molecular mechanisms that regulate access to genetic information remain largely unknown. One recently identified mechanism is based on the physical association of chromatin reader domains with the histone scaffolding proteins that condense the genome within the nucleus of the cell. Essentially, the DNA strands of the genome are spooled around histone octamers (two copies of each histone H2A, H2B, H3, and H4) to form nucleosomes, the basic unit of chromatin. These histone proteins harbor an N-terminal tail that protrudes outside of the core nucleosome (1). The histone tails are readily available for post-translational modifications, such as lysine acetylation and methylation. Methyltransferases modify lysine in a stepwise manner to generate mono- (Kme1), di- (Kme2), or tri-methylated (Kme3) lysine. Each methylation state can be recognized by specific histone mark reader modules and thus lead to divergent biological outcomes. Specifically, reader domains serve as bridges between chromatin and enzymatic activities that open or close the structure of the chromatin, thereby regulating access to genetic information and control gene expression. Aberrant access to genetic information leads to pathologies ranging from cancer to neurological disorders.

Several histone mark readers have been described over the last 15 years, with the chromodomain of the heterochromatin protein HP1α that binds to histone H3 tri-methylated on lysine 9 (H3K9me3) (2) as a prime example. Since then, other reader domains have been discovered, including H3K9me3 readers (ADD, WD40, plant homeodomain, and chromodomains); H3K36me3 readers (tudor, chromobarrel, and PWWP domains); H4K20me2 readers (tandem tudor domain and BAH domain); and H3K4me3 readers (plant homeodomains, tandem tutor domains, double chromodomains, and the zf-CW domain) (3, 4). The plant homeodomain (PHD^7^ finger) is found in several nuclear proteins, but their function was first described in the INhibitor of Growth (ING) family of tumor suppressors. The founding member, p33ING1b, was cloned in a genetic suppressor element screen (5). Then, ING2 (6), ING3 (7, 8), ING4 (9–11), and ING5 (9) were identified, essentially based on sequence similarities with p33ING1b. The PHD finger of ING proteins was shown to mediate interactions with H3K4me3 at the transcriptional start site of genes to stabilize enzymatic activities that regulate access to genetic information (12). Specifically, ING4 binds H3K4me3 at the transcriptional start site to recruit histone acetyltransferase activity and facilitate access to genetic information (13), whereas ING2 bridges histone deacetylase activity to H3K4me3 at transcriptional start site to silence gene expression (14).

ING proteins are broadly lost or mutated in various types of human cancers (15, 16). Overexpression studies suggest their involvement in preventing cellular proliferation, while enhancing cell contact inhibition and DNA damage-induced cell death (12). A comprehensive biochemical characterization of ING proteins demonstrated that p33ING1b and ING2 associate with the mSIN3A-HDAC1 histone deacetylase complex (17, 18), whereas ING3, ING4, and ING5 are found in histone acetyltransferase (HAT) complexes (17). Specifically, ING3 associates with the TIP60 (Tat-interacting protein of 60 kDa) complex (17, 19), ING4 with the HBO1-JADE complex (17), and ING5 with both HBO1-JADE and MOZ-BRPF complexes (17). Thus, p33ING1b and ING2 are believed to primarily function as transcriptional repressors via histone deacetylase activity, whereas ING3, ING4, and ING5 are mainly involved in transcriptional activation via associated HAT activities.

ING3 functions in the multiprotein TIP60 HAT complex, which also includes the ATPases EP400, RUVBL1 (Pontin), and RUVBL2 (Reptin), TRRAP, the bromodomain protein BRD8, the polycomb proteins EPC1 and EPCys-2, the DNA methyltransferase-associated protein DMAP1, actin, and the actin-like protein BAF53A (ACTL6A), the mortality factor MRG15 (MORF4L1), MEAF6, and GAS41 (YEATS4) (17). Interestingly, like ING4 (13), TIP60, RUVBL1, and RUVBL2 are involved in the DNA damage response (20, 21). Moreover, the RUVBL1 and RUVBL2 proteins in the ING3 complex regulate hypoxia signaling much the same as observed with ING4 (22–25), suggesting possible redundant or complementary functions between ING3 and ING4 in the regulation of cellular stress responses. Finally, the components of the ING3 complex described above were recently found to associate with the histone chaperon ANP32E and the histone variant H2AZ (26). Although the role of ING3 in histone exchange remains elusive, ING3 is required along with EPC1 for acetylation of nucleosomes by TIP60 (19).

Importantly, ING2 was found to interact with histone H3. Specifically, the PHD of ING2 binds to H3K4me3 via a conserved aromatic cage (14, 27). The PHD finger-H3K4me3 interaction is critical for both ING2 (14) and ING4 (13) to associate with transcriptional start sites and repress or activate gene expression, respectively. Furthermore, the ING4_PHD_-H3K4me3 interaction was demonstrated to be essential for ING4 tumor suppressive activities, including enhancing DNA damage-induced cell death, inhibition of cellular proliferation, and colony formation (13). Thus, the ability of the PHD finger domain to bridge ING proteins to H3K4me3 is thought to be critical for the tumor suppressor activity of all ING family proteins.

The *ING3* locus is lost or mutated in several human cancers. Notably, frequent loss of heterozygosity is detected at the *ING3* locus, 7q31, in invasive epithelial ovarian carcinomas (28, 29), prostate (30), colorectal (31), as well as human head and neck cancers (8). The 7q31 region contains four candidate tumor suppressor genes, *CAV1*, *CAVal-2*, *ST7*, and *ING3*. Because mutations in the PHD domain of ING3 are also reported in the genomes of various cancers (Cys-376 (frameshift), D380H, and H387P (33)), we decided to investigate the molecular mechanisms that regulate the association of ING3 with histone post-translational modifications. ITC and nuclear magnetic resonance (NMR) experiments demonstrate that the ING3_PHD_ selects for H3K4me3 > H3K4me2 > H3K4me1 > unmodified histone H3 and identified residues critical for ligand coordination. These results are further supported by molecular dynamic (MD) simulation studies of the ING3_PHD_ with modified and unmodified histone H3 and H4 peptides. The trajectory analysis of our MD simulations revealed a conserved aromatic cage composed of residues Tyr-362, Ser-369, and Trp-385, which imparts the affinity and specificity for histone H3 methylated at lysine 4. A structural comparison of the time-averaged structure from the MD simulation of the ING3_PHD_-H3K4me3 complex with x-ray crystal structures of the other ING family member proteins bound to H3K4me3 revealed that the mechanism of histone ligand binding is universally conserved within this protein family. Furthermore, we found that full-length ING3 proteins defective in H3K4me3 recognition are still able to form a complex with the TIP60 HAT, and cell-based assays show that the ING3-H3K4me3 interaction is required for DNA damage-induced cell death. These data illustrate for the first time that histone recognition by the PHD finger region of ING3 is crucial for its activity as a candidate tumor suppressor protein. Interestingly, increased copy number of ING3 along with overexpression and deregulation of ING3 have recently been linked to poor outcomes in prostate cancer patients and castrate-resistant prostate cancer cell lines (34–36). This suggests that ING3 may suppress tumor formation in some cases while promoting cancer in others. Together, our results illustrate the structure and function of the ING3 PHD finger domain in histone recognition and in regulating the biological activity of the ING3 protein, which will be important for the development of new epigenetic therapies aimed a modulating the role of ING3 in disease.

## Results

### 

#### 

##### ING3_PHD_ Recognizes Methylated Histone H3

The ING3 subunit of the TIP60 HAT complex contains a C-terminal PHD finger. PHD finger domains are generally known to recognize methylated lysine on histone tails, and the closely related ING4 and ING5 tumor suppressor proteins have been shown to recognize H3K4me3 through their PHD domains (13, 37, 38). To determine the binding specificity and affinity of the ING3_PHD_, we used a combination of biochemical and biophysical methods to screen the PHD finger against a variety of methylated and unmodified histone tail peptides. We carried out tryptophan fluorescence experiments to test the binding of ING3_PHD_ to histone peptides, H3K4me3 (residues 1–12), H3K4me2 (residues 1–12), H3K4me1 (residues 1–12), H3 unmodified (residues 1–12), and H4 unmodified (residues 1–12). Tryptophan fluorescence is an ideal method to investigate the ING3_PHD_-histone interactions because the binding pocket of the ING3_PHD_ contains two tryptophan residues involved in histone coordination. As seen with other ING PHD finger proteins, ING3_PHD_ preferably recognized histone H3 that was tri-methylated on lysine 4 (*K_D_* = 0.63 μm), and the histone peptide binding affinity decreased in conjunction with the methylation status of lysine 4 (*K_D_* = 4.05 μm for H3K4me2 and 21.45 μm for H3K4me1) (Table 1). Unmodified histone H3 bound the weakest with a binding coefficient of 131.6 μm, and no binding was detected between the ING3_PHD_ and histone H4.

**TABLE 1:** **Dissociation constants of the ING3 PHD finger with post-translationally modified histone peptides as measured by tryptophan fluorescence and ITC**

Histone peptide	Sequence	Trp fluorescence *K_D_*	ITC *K_D_*
		μ*m*	μ*m*
H3K4me3(1–12)	ART**Kme3**QTARKSTG	0.63 ± 0.11	0.93 ± 0.04
H3K4me2(1–12)	ART**Kme2**QTARKSTG	4.05 ± 0.53	2.99 ± 0.33
H3K4me1(1–12)	ART**Kme1**QTARKSTG	21.45 ± 3.51	23.24 ± 0.80
H3 unmodified(1–12)	ART**K**QTARKSTG	131.57 ± 13.48	180.62 ± 19.17
H4 unmodified(4–17)	GKGGKGLGKGGAKR	NA*^a^*	No binding

*^a^* NA, not available.

**FIGURE 1.**
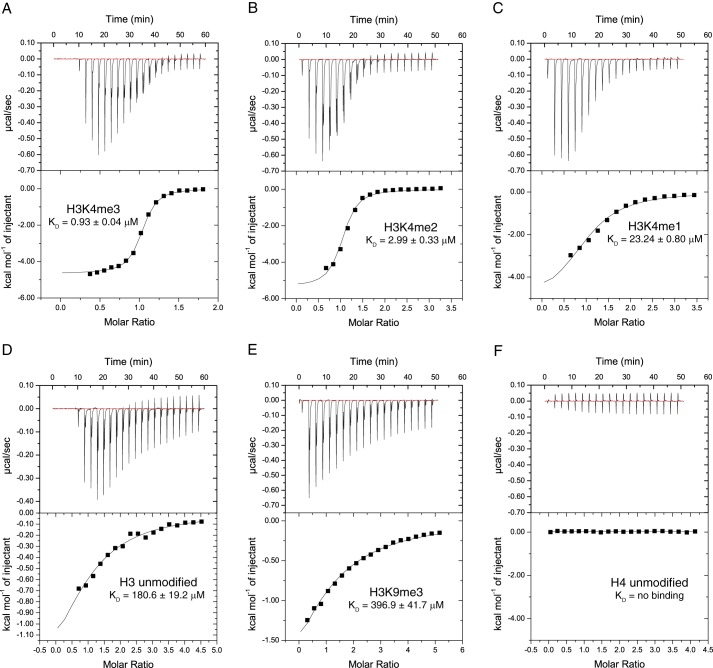
**ITC measurement of the interaction between the wild-type ING3 PHD finger and methylated or unmodified histone peptides.** *A–F,* exothermic ITC enthalpy plots for the binding of the ING3 PHD finger to H3K4me3, H3K4me2, H3K4me1, H3 unmodified, H3K9me3, and H4 unmodified. The calculated binding constants are indicated.

The dissociation constants of the ING3_PHD_ with the histone H3 and histone H4 peptides were also analyzed by ITC experiments (Fig. 1), and the results are included in Table 1. The *K_D_* values determined from the ITC titration data confirmed that the H3K4me3 bound to the ING3_PHD_ with the highest affinity (0.93 μm), followed by H3K4me2 (2.99 μm), H3K4me2 (23.24 μm), and H3 unmodified (180.6 μm), consistent with the tryptophan fluorescence data shown in Table 1. No binding was observed between ING3_PHD_ and the unmodified histone H4 peptide. Our results demonstrate that the ING3 PHD domain preferentially binds to H3K4me3, consistent with the other ING PHD finger proteins (13, 14, 27, 37, 39).

##### Chemical Shift Mapping of the ING3 Binding Pocket

**FIGURE 2.**
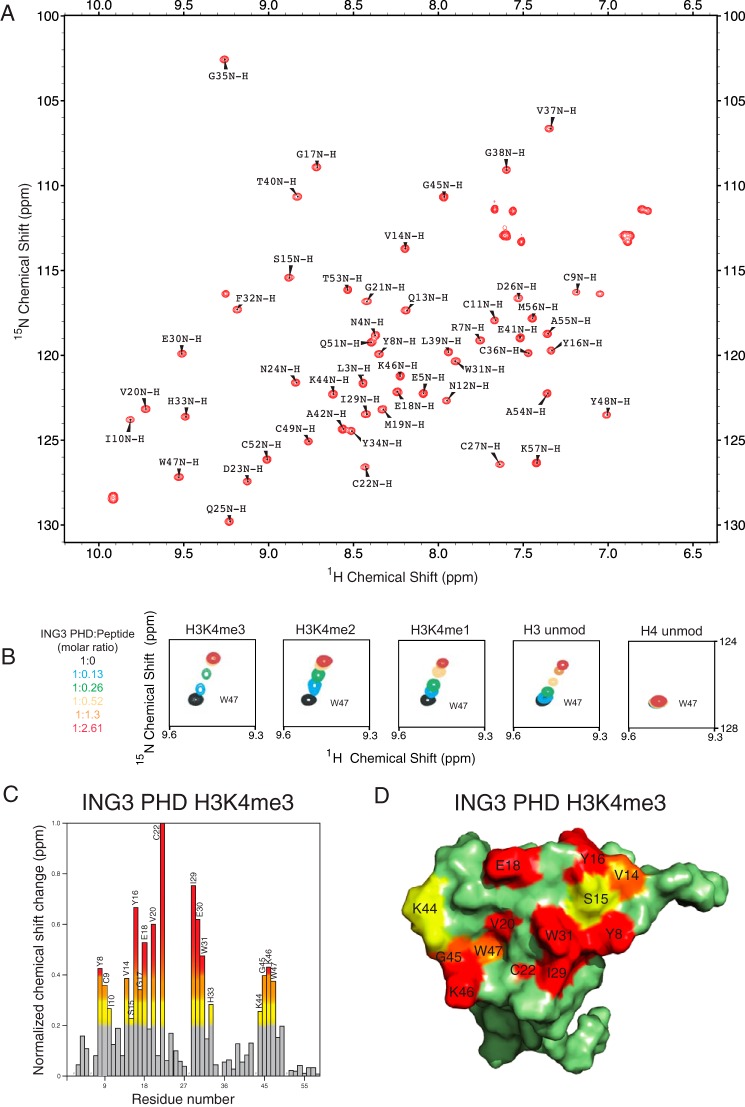
**Interaction of the ING3 PHD finger with histone ligands.** *A,* two-dimensional ^1^H-^15^N HSQC spectra of ^15^N-labeled ING3 PHD finger with the complete HSQC assignments labeled. *B,* superimposed ^1^H-^15^N HSQC spectra of the 0.5 mm ING3 PHD finger, collected during titrating in the indicated histone peptides. The spectra are color-coded according to the protein/peptide ratio. *C,* histogram of normalized ^1^H-^15^N chemical shift changes in backbone amides of the ING3 PHD finger upon addition of the H3K4me3 peptide. Chemical shift changes were from 0.2 to 0.3 ppm (*yellow*), from 0.3 to 0.4 ppm (*orange*), and >0.4 ppm (*red*). *D,* mapping of residues exhibiting significant resonance perturbations upon addition of the H3K4me3 ligand onto the surface of the NMR structure of the ligand-free ING3 PHD finger (PDB code 1X4I). The residues in the binding pocket are colored *red, orange,* and *yellow* depending on the magnitude of the chemical shift change upon ligand addition as in *C*.

To outline the specific interactions between the histone peptide ligands and the ING3_PHD_ binding pocket, we carried out nuclear magnetic resonance (NMR) experiments. The backbone assignments of the ING3_PHD_ finger were obtained from the ^15^N,^13^C double-labeled ING3_PHD_ using the ADAPT-NMR program at the NMRFAM facility in Madison, WI, which allowed for rapid data collection and assignment of the NMR spectra (Fig. 2*A*). To confirm recognition of the histone tail peptides observed by tryptophan fluorescence and ITC, the ^1^H-^15^N heteronuclear single quantum coherence (HSQC) spectra of the uniformly ^15^N-labeled ING3 PHD finger were recorded in the absence and presence of the following histone tail peptides: unmodified histone H3 (residues 1–12), unmodified histone H4 (residues 4–17), and H3K4me1, H3K4me2, and H3K4me2 (residues 1–12). Chemical shift perturbations were induced in the ING3_PHD_ upon addition of unmodified H3, H3K4me1, H3K4me2, and H3K4me3 (Fig. 2*B*). No significant resonance shifts were observed upon the addition of unmodified histone H4 peptide. This pattern of histone recognition is similar to the recognition of the histone tails by other ING PHD finger proteins (13, 37) and confirms a strong interaction of the ING3_PHD_ domain with methylated histone H3 tails.

**FIGURE 3.**
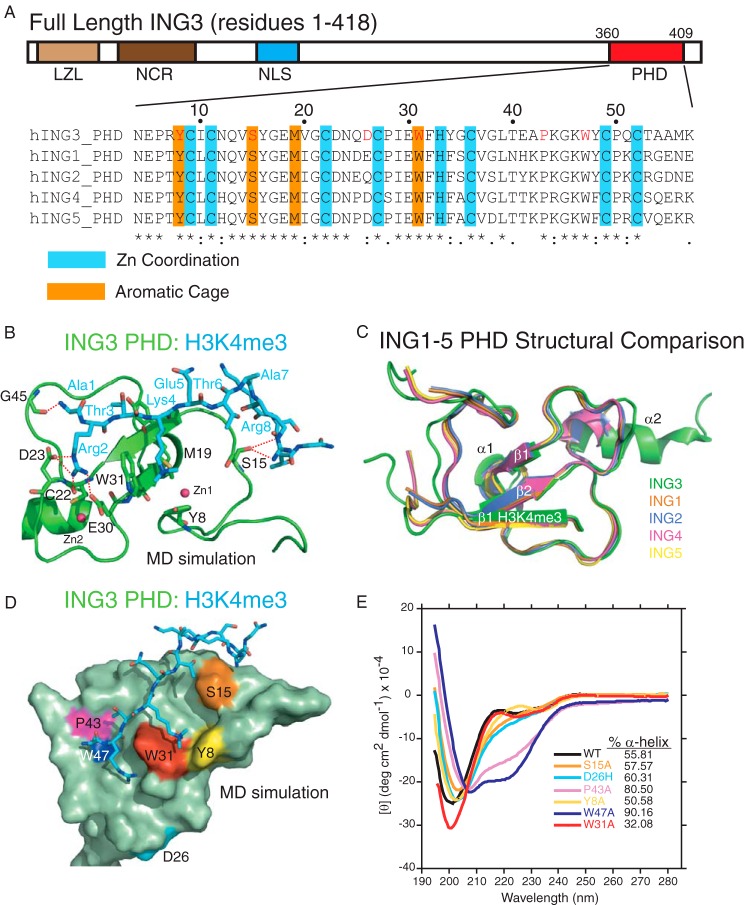
**Characterization of specific ING3 PHD finger-histone ligand interactions.** *A,* domain architecture of the full-length human ING3 protein with the sequence alignment of the PHD finger domains of ING1–5. Residues 1–418 of human ING3 are shown with the N-terminal leucine zipper-like domain (*LZL*) and novel conserved region (*NCR*), as well as the nuclear localization signal (*NLS*) and C-terminal PHD. Sequence alignment of the PHD finger domains of ING1–5 were aligned to the ING3_PHD_ construct used in this study (residues 4–57, corresponding to residues 360–409 in full-length ING3). The conserved Cys_4_-His-Cys_3_ PHD finger motif residues involved in zinc coordination are highlighted in *blue*; the aromatic cage residues are highlighted in *orange,* and residues mutated in this study are colored in *red. B,* time-averaged structure of the ING3 PHD finger in complex with the H3K4me3 peptide ligand obtained using MD simulation. PyMOL (32) was used to depict the ING3 PHD finger, shown in *green*, and the peptide ligand is colored in *blue*. Hydrogen bonds and salt bridges are indicated by *red dotted lines. C,* structural alignment of the ING1–5 PHD finger proteins in complex with the H3K4me3 histone peptide. Structures of the ING PHD complexes were taken from the Protein Data Bank and aligned in PyMOL (32). The structures include ING1 (2QIC, *orange*), ING2 (2G6Q, *blue*), ING3 (MD simulation, *green*), ING4 (2PNX, *magenta*), and ING5 (3C6W, *yellow*). *D,* surface representation of the time-averaged structure of the ING3_PHD_-H3K4me3 complex from the MD simulation showing specific point mutations introduced into the binding pocket by site-directed mutagenesis, with the H3K4me3 histone ligand in the binding pocket shown in *blue. E,* circular dichroism spectra in the far-UV region of the ING3 PHD finger wild-type and mutant proteins. The percent α-helical content of each protein is listed in the *inset*.

The chemical shift perturbations observed upon binding of the H3K4me3 histone peptide were used to map the binding pocket of the ING3 PHD finger. The normalized changes in chemical shift from the NMR HSQC spectrum were plotted as a bar graph to show the amino acid residues most affected by addition of the H3K4me3 histone tail peptide in a 1:2.61 ING3_PHD_-to-peptide ratio. The largest chemical shifts (changes greater than 0.4 ppm) are shown in *red*, and changes of >0.3 or >0.2 ppm are shown in *orange* and *yellow*, respectively (Fig. 2*C*). Nine amino acids within the ING3_PHD_ showed large chemical shift changes upon addition of the H3K4me3 histone ligand, including Tyr-8, Tyr-16, Glu-18, Val-20, Cys-22, Ile-29, Glu-30, Trp-31, and Lys-46, indicating that these residues are directly or indirectly involved in ligand binding. Mapping the position of these amino acids onto the surface of the native ING3_PHD_ structure (PDB code 1X4I) reveals that there are two adjacent ligand coordination regions on the surface of the PHD finger (Fig. 2*D*). The first group of residues with large chemical shift changes is clustered around Trp-31, which makes up the side of the aromatic cage responsible for coordination of the tri-methylated lysine in other PHD finger proteins, including ING4 and ING5 (13, 37, 40). The second group of residues is located near Trp-47 and comprises a potential binding site for the N terminus of the histone H3 tail, which in the ING5_PHD_ (PDB code 3C6W) structure was a region critical for ligand recognition and coordination (37). Notably, the residues showing the largest chemical shift changes in the PHD binding pocket are conserved with the ligand-coordinating residues observed in the ING4 and ING5 crystal structures bound to H3K4me3 (13, 37, 40) (also see the sequence alignment in Fig. 3*A*). These include Trp-47, Tyr-8, and Ser-15, which make up the aromatic cage in the ING5_PHD_ structure (37). Residue Cys-22, which shows the largest chemical shift change of any residue, is one of the conserved cysteines responsible for coordinating one of the two zinc ions in the ING3_PHD_ finger. In the ING5_PHD_ structure, the residue conserved with Cys-22 (Cys-202) makes an important hydrogen bond contact to Arg-2 in the H3K4me3 histone tail peptide, and our NMR chemical shift data (in combination with our MD simulation data, Fig. 3*B*) indicate that Cys-22 likely makes a large conformational change moving from a more buried position to the surface of the ING3_PHD_ binding pocket upon ligand binding.

##### Molecular Dynamic (MD) Simulations of Histone Ligand Binding

Because our attempts to crystallize the H3K4me3 histone peptide in complex with the ING3 PHD finger were unsuccessful, we used MD simulations to further investigate the ING3_PHD_-histone ligand interactions. The simulations were carried out for the experimentally studied peptides bound to ING3 to correlate differences in the binding affinities measured among histone ligands and specific molecular interactions observed in the protein-ligand complexes. All the peptides used in MD simulations were protonated on their N terminus (NH_3_^+^) and amidated on the C terminus to represent the structures of the peptides used in the *in vitro* experiments. The initial MD simulation structures of the methylated histone H3 peptides in complex with the ING3_PHD_ protein were obtained by flexible docking of each of the four histone peptides listed in Table 1 to the ING3_PHD_ using the FlexPepDock program (41, 42) from Rosetta commons (43). The initial approximate binding poses for FlexPepDock were taken from the x-ray crystal structures of the ING4_PHD_ and ING5_PHD_ proteins in complex with H3K4me3 peptides (PDB codes 2PNX and 3C6W, respectively). Both ING4_PHD_ and ING5_PHD_ share very high sequence and structural identities with the ING3_PHD_, especially among the binding site residues (Fig. 3*A*) (40). Because of the absence of experimental structures for related PHD finger proteins bound to the unmodified histone H4 peptide, the initial FlexPepDock binding pose for this ligand was obtained by docking histone H4 using the Hex webserver (44), which does not require an initial approximate bound conformation of the peptide. Then docking of the histone H4 peptide was refined in FlexPepDock, so that the top scored complex of each peptide generated with FlexPepDock could be used as the starting point for MD simulations.

The MD simulations, which were run using the Amber14 package for 10 ns under isobaric and isothermal conditions, provided detailed insights into the molecular interactions between the ING3_PHD_ and its histone ligands (Tables 2 and 3). The ING3_PHD_ domain is a typical PHD finger fold with two structural Zn^2+^ atoms that are coordinated by a conserved pattern of cysteine and histidine residues (Zn1-CCCC and Zn2-CCCH) with tetrahedral geometry. Coordination of the zinc ions by the Cys_4_-His-Cys_3_ consensus sequence stabilizes the three loop regions of the PHD finger and is essential for folding of secondary structures, including the double-stranded antiparallel β-sheet that forms the core of the PHD domain (Fig. 3*B*). The structural integrity and secondary structural elements of the protein were maintained well throughout the simulations, which was ascertained by the coordination geometries measured between the Zn^2+^ cations and interacting residue atoms, and the secondary structure analysis (data not shown).

**TABLE 2:** **ING3-histone peptides binding energies by free energy calculations**

No.	Peptide	Binding free energies and its components (kcal/mol)				
		Δ*S**^a^*	MM/GBSAb*^b^*		MM/PBSAb*^b^*	
			GBSA Δ*G*	Δ*G**^c^*	PBSA Δ*G*	Δ*G**^c^*
	*kcal/mol*					
1	H3K4me3	−42.358	−70.332	−27.975	−65.248	−22.890
2	H3K4me2	−40.958	−62.682	−21.724	−61.206	−20.249
3	H3K4me1	−42.956	−60.704	−17.749	−61.068	−18.112
4	H3K4me0	−38.316	−56.285	−17.970	−54.131	−15.816
5	H4-unmodified	−39.460	−42.744	−3.285	−43.705	−4.245

*^a^* Rotational, translational, and vibrational entropy was from normal mode analysis.

*^b^* Molecular Mechanics/Generalized Born Surface Area and Molecular Mechanics/Poisson-Boltzmann Surface Area Methods are described in Ref. 45.

*^c^* Difference between GBSA or PBSA Δ*G* and Δ*S* is shown.

**TABLE 3:** **Hydrogen bond interactions between the ING3 PHD finger and histone peptides from MD simulations**

Histone peptide	Interacting residues		% present	Average bond length	Average bond angle
	Peptide	ING3			
				Å	°
H3K4me3	K4me3@N-H	Met-19@O	99.96	2.95	159.85
	K4me3@O	Met-19@N-H	98.44	2.93	153.88
	Arg-8@O	Gly-17@N-H	99.46	2.96	159.71
	Thr-6@N-H	Gly-17@O	84.08	3.09	150.95
	Arg-2@O	Gly-21@N-H	99.10	2.99	155.59
	Arg-2@NH_2_-HH21	Cys-22@O	69.38	3.02	141.96
	Arg-2@NH_2_-HH22	Glu-30@OE2	50.86	2.89	150.60
	Arg-2@NH1-HH11	Glu-30@OE2	50.64	2.93	148.85
	Arg-2@NH1-HH12	Glu-30@OE1	46.54	2.86	152.13
	Arg-8@NE-HE	Ser-15@O	85.44	2.97	145.35
H3K4me2	K4me2@N-H	Met-19@O	99.86	3.01	157.29
	K4me2@O	Met-19@N-H	97.06	2.94	150.87
	Arg-8@O	Gly-17@N-H	99.46	2.95	159.54
	Arg-2@O	Gly-21@N-H	99.34	2.98	154.54
	Arg-2@NE-HE	Asp-23@OD1	77.36	2.88	149.88
	Arg-2@NH_2_-HH21	Asp-23@OD1	77.10	2.84	148.06
	Thr-6@N-H	Gly-17@O	78.76	2.93	151.51
H3K4me1	K4me2@N-H	Met-19@O	99.88	2.98	157.16
	K4me2@O	Met-19@N-H	98.24	2.93	154.48
	Arg-2@NE-HE	Asp-23@OD1	72.02	2.86	150.64
	Arg-2@O	Gly-21@N-H	99.22	3.02	155.08
	Arg-2@NH1-HH12	Glu-30@OE1	77.96	2.83	153.52
	Arg-2@NH_2_-HH22	Glu-30@OE1	76.02	2.98	143.27
	Arg-2@NH_2_-HH21	Asp-23@OD1	64.78	2.94	142.04
	Arg-8@O	Gly-17@N-H	58.44	3.04	159.48
	Thr-6@N-H	Gly-17@O	56.62	3.01	149.52
H3 unmodified	Lys-4@NH	Met-19@O	99.36	2.95	155.64
	Lys-4@O	Met-19@N-H	92.94	2.98	155.10
	Arg-2@O	Gly-21@N-H	98.50	2.99	152.15
	Arg-8@O	Gly-17@N-H	97.54	2.99	158.64
	Arg-2@NE-HE	Asp-23@OD1	72.08	2.89	149.81
	Arg-2@NH_2_-HH21	Asp-23@OD1	69.96	2.86	147.77
	Thr-6@N-H	Gly-17@O	59.72	3.12	151.08
H4 unmodified	Gly-3@N-H	Ser-6@O	83.62	3.06	151.85
	Gly-4@O	Tyr-8@N-H	49.70	3.21	142.92
	Gly-3@N-H	Ser-3@O	42.78	2.96	151.46

The MD simulation data were processed using molecular mechanics/Poisson-Boltzmann surface area (MM/PBSA) and Generalized Born surface area (MM/GBSA) formalisms (45), utilizing the normal-mode analysis to obtain rotational, translational, and vibrational entropy (46). The resulting binding free-energy values (Δ*G*) and their components are summarized for the histone peptides in Table 2. The relative energies calculated for the histone peptides (H3K4me3, H3K4me2, H3K4me1, H3 unmodified, and H4 unmodified) show good agreement with the experimental NMR, ITC, and Trp fluorescence binding data. The most favorable binding energy was observed for the H3K4me3 peptide, followed by the H3K4me2, H3K4me1, and H3 unmodified peptides, respectively. The non-binding histone H4 unmodified peptide correctly generated a relatively weak binding energy. The trajectories from each of 10-ns MD simulations (a total of 5000 snapshots or frames, each representing a 2-ps time interval) were analyzed for the presence of H-bond interactions between the ING3_PHD_ protein and the histone peptide residues. The strength and geometries were quantitatively described in terms of bond length and donor-H-acceptor bond angles as well as the longevities of the H-bond as % of frames (5000 frames represent 100%), in which the bonds were seen.

In the time-averaged structure of the ING3_PHD_-H3K4me3 complex from the MD simulation (Fig. 3*B* and supplemental data file ING3PHD-H3K4me3-MDsimulation.pdb), the histone peptide binds to the PHD domain forming a third antiparallel β-strand that pairs with the central β-sheet core described above (Fig. 3*C*). The most common interactions seen with all of the methylated histone H3 peptides (mono-, di-, and tri-methylated and non-substituted lysines) and the ING3_PHD_ are intra-β-strand backbone contacts between the Lys-4 residues of the histone peptides and Met-19 in ING3_PHD_. Table 3 summarizes the hydrogen bond interactions seen at least 40% of the time throughout the production phase of MD simulation. The average bond lengths and bond angles of these H-bonds are also summarized in Table 3. For example, the H-bonds between backbone atoms of Met-19 and Gly-21 of ING3 and the histone H3 peptide residues Lys-4 and Arg-2 occur for nearly the entire 10-ns trajectory of all MD simulations (92.9–99.9%). These conserved intra-β-sheet hydrogen bonds facilitate the important cation-π interactions exhibited by the methylated histone peptides and residues Trp-31 and Tyr-8 in the ING3_PHD_.

As in other ING PHD finger proteins, the H3K4me3 peptide binds in a deep and extensive pocket that consists of two large grooves connected by a narrow channel (37, 40). The tri-methylated Lys-4 side chain fits into one groove containing residues Trp-31, Tyr-8, and Ser-15 identified from the NMR chemical shift perturbation and MD simulation data (Figs. 2*D* and 3*B*). Fig. 3*B*, highlights the hydrogen bond interactions observed between amino acid side chains of the histone H3K4me3 peptide and the ING3_PHD_. For instance, the side chain amino group of Arg-2 in the histone peptide makes a constant H-bond interaction with the side chain carboxylic group of Glu-30 of ING3_PHD_, which was observed throughout the entire 10-ns simulation time (Table 3). Table 3 includes the details of the Arg-2 interaction from the MD simulations and also reveals several strong H-bond interactions made by the Thr-6 and Arg-8 residues of H3K4me3.

##### Molecular Mechanism of Methyl-lysine Coordination

**FIGURE 4.**
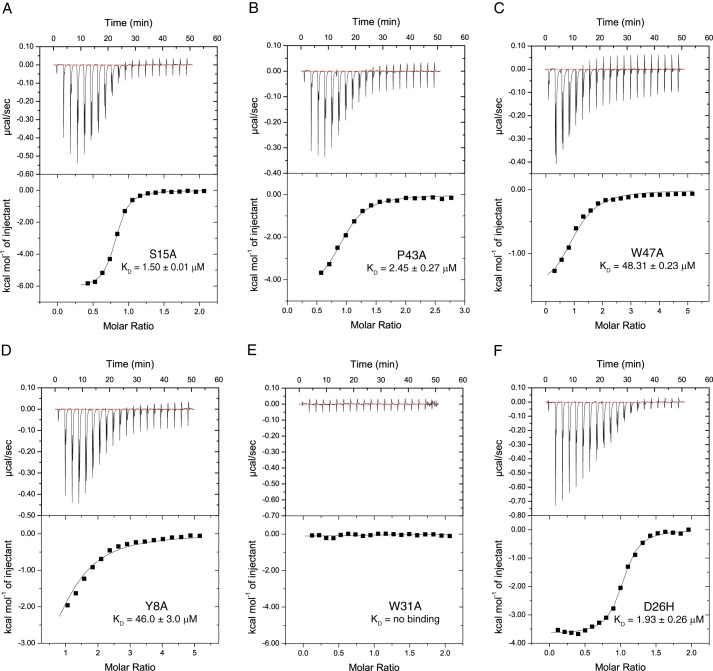
**ITC measurements of interactions between mutant ING3 PHD finger proteins and the H3K4me3 histone tail ligand.** *A–F,* exothermic ITC enthalpy plots for the binding of the ING3 PHD finger mutant proteins to the H3(1–12)K4me3 peptide. The *inset* lists the measured binding constants.

Our NMR chemical shift perturbation experiments and MD simulation data of the ING3_PHD_-H3K4me3 ligand complex suggest that several amino acids in the ING3_PHD_ binding pocket are crucial for histone recognition and coordination of the tri-methylated lysine moiety. To further investigate the contribution of specific amino acids to ligand binding, we carried out site-directed mutagenesis on ING3_PHD_ residues and measured their effect on ligand binding affinity with ITC experiments. We selected ING3_PHD_ residues Trp-47, Ser-15, Pro-43, Tyr-8, and Trp-31 for mutagenesis to alanine because of their proximity to the binding pocket and/or direct involvement in H3K4me3 coordination (Fig. 3*D*). As shown in Table 4 and Fig. 4, the W31A mutation had the largest effect on histone peptide binding, resulting in no recognition of this ligand. This is likely due to the importance of residue Trp-31 in formation of the aromatic cage in the ING3_PHD_ as well as cation-π interactions between the side chain of this residue and the trimethylammonium group of Lys-4. The W47A and Y8A mutations reduced the ING3_PHD_ binding affinity from 0.93 μm for the WT protein to 48 and 46 μm in the mutant proteins, respectively. This observation is not surprising because Trp-47 forms the bottom of the Arg-2 binding pocket and Tyr-8 comprises one side of the aromatic cage around Lys-4. Both Trp-47 and Tyr-8 likely contribute to the structural features of the binding pocket and make important hydrophobic interactions with the histone ligands. Additionally, mutation of Trp-47 to alanine resulted in significant structural changes in the ING3_PHD_ causing a large increase in the α-helical content of the protein as observed by circular dichroism (Fig. 3*E*). These results indicate that Trp-47 also plays a role in the hydrophobic packing and folding of the ING3 PHD finger motif. Mutation of ING3_PHD_ residues Pro-43 and Ser-15 had a less dramatic effect, resulting in a 2–3-fold drop in binding affinity for the H3K4me3 histone ligand (Table 4). Pro-43 was previously shown to be important for the coordination of Ala-1 of the histone H3 peptide for ING5_PHD_ (37); however, there were no hydrogen bond interactions observed in any of our MD simulations (Table 3). Interestingly, the P43A mutation did change the α-helical content of the ING3_PHD_ from 55 to 80%, indicating it does play an important structural role in protein folding/packing. Residue Ser-15 is highly conserved in all ING PHD finger proteins and makes up one side of the Lys-4 binding pocket. However, mutation of Ser-15 to alanine only produced a 2-fold drop in ligand binding affinity. The trajectory analysis of the MD data indicates that in the ING3_PHD_-H3K4me3 complex, Ser-15 does make hydrogen bond contacts to the Arg-8 side chain in the histone peptide over 85% of the simulation time (Table 3), but the modest 2-fold drop in binding affinity observed by ITC suggests that this interaction is less critical for histone ligand coordination than Tyr-8 and Trp-47 (Table 4). This is corroborated by only moderate chemical shift changes of the Ser-15 residue upon addition of the H3K4me3 peptide in NMR titration experiments (Fig. 2*C*).

**TABLE 4:** **Binding of the H3K4me3 peptide by ING3 PHD mutants by ITC**

Mutant	ITC *K_D_*
	μ*m*
ING3 PHD S15A	1.50 ± 0.01
ING3 PHD D26H	1.93 ± 0.26
ING3 PHD P43A	2.45 ± 0.27
ING3 PHD Y8A	46 ± 2.99
ING3 PHD W47A	48.31 ± 0.23
ING3 PHD W31A	No binding

Because the ING3 protein is a known tumor suppressor that is down-regulated in multiple cancers (8, 47, 48), we searched the cBioPortal for Cancer Genomics to see whether any mutations in the PHD finger of ING3 have been reported in cancer patients (33, 49). Our search uncovered three mutations in the ING3_PHD_, which include a frameshift insert at Cys-376 (Cys-22 in our MD simulation structure) found in cutaneous skin melanoma, a missense mutation H387P (His-33) associated with bladder carcinoma, and a missense mutation at D380H (Asp-26) found in head and neck carcinoma. Because the Cys-22 and His-33 residues are important for zinc ion coordination in the PHD finger motif, and their mutation would likely result in unfolding of the PHD domain, we focused on the effect of the D26H mutation on histone recognition by the ING3_PHD_. As with the S15A and P43A mutations, the D26H mutation resulted in a slightly weaker binding affinity for the H3K4me3 histone peptide as compared with the WT ING3_PHD_ with a *K_D_* of 1.93 ± 0.26 μm (Table 4 and Fig. 4*F*). Interestingly, according to our circular dichroism analysis, this mutation increases the α-helical content slightly, from 56% in the WT ING3_PHD_ to 60% in the ING3_PHD_-D26H protein (Fig. 3*E* and Table 5). As seen in Fig. 3*D*, residue Asp-26 (highlighted in *cyan*) is located peripheral to the histone binding pocket, so it is not surprising that this mutation has only a minor effect on histone recognition.

**TABLE 5:** **Analysis of ING3 PHD finger proteins by circular dichroism**

ING3 PHD protein	α-Helix	β-Strand
	%	%
WT	55.81	1.85
S15A	57.57	0.99
D26H	60.31	0.81
P43A	80.5	0.06
Y8A	50.58	2.57
W47A	90.16	0.04
W31A	32.08	5.44

##### Interaction of ING3 with the TIP60 Complex

**FIGURE 5.**
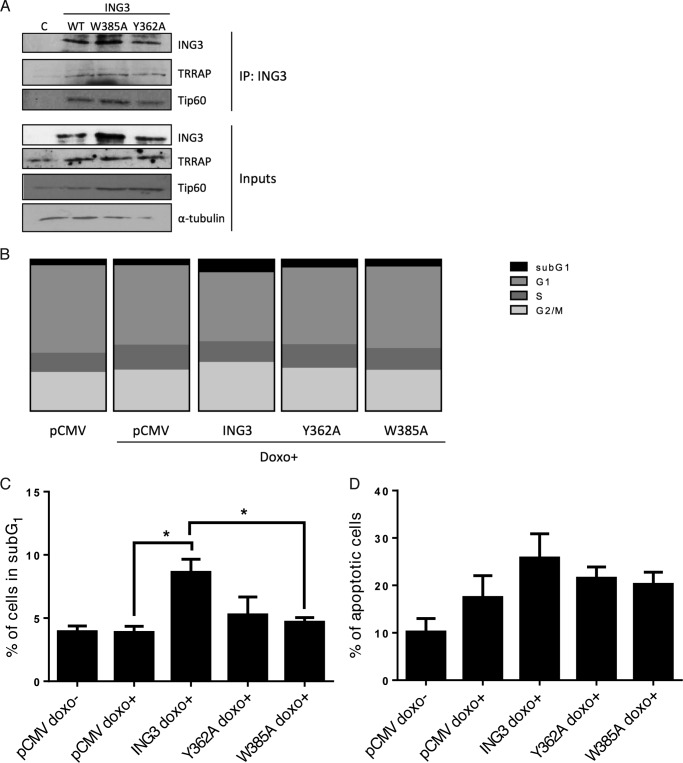
**ING3 mutant proteins form a complex with TIP60, and the ING3_PHD_-H3K4me3 association is required for ING3-induced DNA damage-dependent cell death.** *A,* full-length ING3 mutant proteins defective in H3K4me3 binding were retained within the TIP60 complex. COS-7 cells were transfected with pCMV-3×FLAG-ING3 wild-type (WT), Y362A (Y362A), or W385A (W385A) vectors for 72 h. Exogenous ING3 was immunoprecipitated with a FLAG antibody followed by immunoblotting. Empty pCMV-3×FLAG vector was used as a control in *lane C. B* and *C,* MCF7 cells were stably transduced with FLAG-ING3-expressing retroviral particles as indicated for 72 h, and for the final 20 h cells were treated with 400 ng/ml doxorubicin (*doxo*^+^). Subsequently, cell cycle status was determined by flow cytometry analysis of propidium iodide staining. Distribution of cells in cell cycle phases (*B*) and percentage of cells in sub-G_1_ phase (*C*) were determined. Data were plotted as the mean ± S.E. of three independent experiments; statistical analysis was undertaken with *t* test, and the * indicates *p* < 0.05. *D,* MCF7 cells were transduced with retroviral particles as in *B* for 72 h, and for the final 20 h the cells were treated with 400 ng/ml doxorubicin (*doxo*^+^). Subsequently, the percentage of apoptotic cells was determined by flow cytometry detection of annexin V-positive cells. Data were plotted as the mean ± S.E. of three independent experiments, and statistical analysis was undertaken with *t* test.

As described above, the ING3 protein is a functional subunit of the TIP60 HAT, and recognition of methylated histones by the ING3_PHD_ is thought to recruit this multisubunit chromatin remodeling complex to histones to regulate gene expression (17). To determine whether ING3 mutants defective in H3K4me3 binding were retained within the TIP60 HAT complex (17, 19), FLAG-tagged full-length (residues 1–418) ING3_WT_, ING3_Y362A_, and ING3_W385A_ forms were expressed in highly transfectable COS-7 monkey cells. Then, anti-FLAG immunoprecipitates were analyzed by immunoblotting with anti-TIP60 and anti-TRRAP antibodies. As observed with ING2 (14, 50) and ING4 (13), the defective H3K4me3-binding mutants, ING3_Y362A_ and ING3_W385A_ proteins do associate with the TIP60 and TRRAP subunits of the HAT complex (Fig. 5*A*).

The exogenous expression of ING proteins generally potentiates DNA damage responses and induces apoptosis (13). To determine whether the association with H3K4me3 is required for ING3 to induce apoptosis, human mammary carcinoma MCF7 cells were transduced with the ING3_WT_, ING3_Y362A_, or ING3_W385A_ proteins (which correspond to residues Tyr-8 and Trp-31 highlighted in the time-averaged structure of the ING3_PHD_-H3K4me3 complex from the MD simulation, Fig. 3*D*), and DNA damage was induced by treating the cells with doxorubicin. Expression of ING3_WT_ affected the cell cycle, and after treatment with doxorubicin we observed a decreased percentage of cells in both G_1_ and S phase (Fig. 5*B*). Interestingly, doxorubicin induced an increase in the sub-G_1_ population in the presence of ING3_WT_ (*p* value 0.005), but not with the ING3_Y362A_ or ING3_W385A_ forms (Fig. 5, *B* and *C*), suggesting that the association of ING3 with H3K4me3 is required for the full-length ING3 protein to regulate apoptosis. Indeed, in parallel experiments, addition of doxorubicin induced an increase in annexin V staining in ING3_WT_-expressing cells (*p* value 0.006) but not in ING3_Y362A_- or ING3_W385A_-expressing cells (*p* values of 0.2 and 0.3, respectively) (Fig. 5*D*). These results are consistent with functional studies on other ING proteins, which suggest that H3K4me3 recognition tethers ING3 to its histone ligands and stimulates its biological activity (13, 14, 27, 37, 39).

## Discussion

Our results demonstrate that the ING3 PHD finger preferentially binds to histone H3K4me3 over H3K4me2, H3K4me1, and the unmodified histone H3. Furthermore, this study confirms that selection of tri-methylated histones by the ING3_PHD_ is a conserved function of the PHD finger domains in all ING family proteins, including ING1, ING2, ING4, and ING5, which underscores its importance in directing their biological function (13, 14, 27, 37, 39). The molecular mechanism of histone recognition by the ING3 PHD finger was evaluated using experimentally observed interactions between the ING3_PHD_ and the histone H3K4me3 ligand with a combination of NMR chemical shift perturbation experiments and mutational analysis. The experimental results are supported by data obtained from the time-averaged structure of the ING3_PHD_-H3K4me3 complex using MD simulation. The Trp-31 and Tyr-8 residues help form an aromatic cage in the ING3_PHD_ binding pocket, and they directly coordinate the tri-methylated lysine 4 in the histone H3 ligand. To recognize and specifically select for histone H3, ING3_PHD_ also makes important contacts using residues Trp-47, Asp-23, and Cys-22 to create a secondary binding pocket and to form hydrogen bonds to Arg-2 in the histone peptide. As observed with other PHD fingers, the two grooves in the ING3_PHD_ H3K4me3 binding pocket that coordinate Lys-4 and Arg-2 are separated by a narrow channel, which precludes binding of histone peptides with a large side chain at position 3. MD simulations revealed that the trimethylammonium group of Lys-4 is largely coordinated by hydrophobic and cation-π interactions with residues Tyr-8 and Trp-31 in the ING3_PHD_ binding pocket. Additionally, mutation of Tyr-8 and Trp-31 prevented histone binding in our ITC assays. These results demonstrate that formation of the ING3-H3K4me3 complex is driven by a combination of hydrogen bonding, complementary surface interactions, and hydrophobic contacts.

We used the time-averaged structure of the ING3_PHD_-H3K4me3 complex generated by MD simulation to compare coordination of the histone H3K4me3 peptide by ING3_PHD_ with x-ray crystal structures of the other ING PHD fingers bound to H3K4me3. Superposition of the ING3_PHD_-H3K4me3 MD simulation onto the ING1_PHD_, ING2_PHD_, ING4_PHD_, and ING5_PHD_ structures revealed that the overall structural fold and the histone binding mechanism among these PHD fingers is highly conserved (Fig. 3*C*). The time-averaged structure of the ING3_PHD_-H3K4me3 complex from the MD simulation is most closely related to the x-ray crystal structure of ING1_PHD_ (PDB code 2QIC, r.m.s.d. of 0.86 Å over 53 Cα atoms), followed by ING2_PHD_ (PDB code 2G6Q, r.m.s.d. of 0.87 Å), ΙNG5_PHD_ (PDB code 3C6W, r.m.s.d. 1.094 Å), and ING4_PHD_ (PDB code 2PNX, r.m.s.d. 1.098 Å). Interestingly, our ligand-bound time-averaged MD simulation structure of the ING3_PHD_-H3K4me3 complex superimposes more closely with the other ligand-bound ING PHD finger structures than it does with the ligand-free NMR structure of ING3_PHD_ (PDB code 1X4I) (r.m.s.d., range from 1.3 o 2.0 Å over the Cα atoms) (40). As seen in Fig. 3*C*, the orientation of the H3K4me3 histone peptide in the binding pocket is conserved among all five of the ING_PHD_-H3K4me3 structures. In addition, the same specific molecular interactions observed in our MD simulation of ING3_PHD_-H3K4me3 complex are also found in the other ING_PHD_-H3K4me3 structures, revealing that all of the ING PHD fingers use a conserved binding mode for histone ligand recognition. For example, in the time-averaged structure of the ING3_PHD_-H3K4me3 complex from the MD simulation, the semi-aromatic cage is formed around the trimethylammonium group of Lys-4, in which Trp-31 and Tyr-8 make cation-π, hydrophobic, and van der Waals contacts with this group. Residues Met-19 and Ser-15 compose the remainder of the hydrophobic cage, and all four of these residues are conserved in the PHD finger binding pockets of ING1–5 (Fig. 3*A*) (40). Additionally, the coordination of Arg-2 in the H3K4me3 histone peptide is similar among the ING PHD finger proteins with hydrogen bond contacts between the side chains of Glu-30, Asp-23, and the backbone carbonyl of Cys-22 in ING3_PHD_, to the side chain amino groups of Arg-2 (Fig. 3*B*). The same interactions are observed between residues Glu-234, Asp-227, and Cys-226 of the ING1 PHD finger and the Arg-2 side chain in the H3K4me3 histone peptide (39), and this bonding pattern is also seen in the ING2, ING4, and ING5 PHD finger proteins (13, 27, 37). Thus, this structural comparison of the molecular recognition of H3K4me3 by the ING3 PHD finger with other ING_PHD_ proteins reveals that the mechanism of histone ligand binding is universally conserved within the ING protein family.

Finally, experiments with the full-length ING3 protein revealed that PHD finger mutations that inhibit H3K4me3 binding do not have an effect on the presence of ING3 in the TIP60 HAT complex, but they do prevent ING3 from up-regulating DNA damage-induced cell death. A recent study also showed that the chromodomain in TIP60 recognizes H3K9me3 and stimulates the acetylation of the ataxia telangiectasia mutated protein kinase in DNA double strand break repair (20). The recruitment of large enzymatic complexes involved in chromatin remodeling is often carried out by epigenetic “reader” domains that recognize post-translational modifications on the nucleosomes (37, 51–54). These data indicate that recognition of H3K4me3 by the ING3 PHD is necessary to target the TIP60 complex acetyltransferase activity to up-regulate apoptosis. Collectively, the structural and functional information presented here will be essential for further study of the biological activity of ING3 in the TIP60 HAT complex and the role of the ING3 PHD finger domain in epigenetic signaling by this complex.

## Experimental Procedures

### 

#### 

##### ING3 PHD Plasmid Construction

The ING3 PHD finger region (residues 360–409) was amplified using PCR and cloned into the pDEST15 vector encoding an N-terminal glutathione transferase (GST) tag using the Gateway cloning technology (Invitrogen) as described previously (55). ING3_PHD_ proteins with single mutations at W31A, W47A, S15A, P43A, Y8A, and D26H were generated using the QuikChange® mutagenesis procedure (Stratagene) as described previously (56). The DNA sequence for wild-type and mutant proteins was verified at the University of Vermont DNA facility, and the plasmids were transformed into in *Escherichia coli* Rosetta^TM^ 2(DE3)pLysS competent cells (Novagen) for protein expression.

##### ING3 PHD Finger Expression and Purification

*E. coli* cells containing the wild-type GST-tagged ING3_PHD_ were grown in Terrific Broth (TB) or in ^15^NH_4_Cl-supplemented or ^15^NH_4_Cl/^13^C_6_ d-glucose-supplemented minimal media. The cultures were grown at 37 °C to an *A*_600_ of 1, induced with 0.25 mm isopropyl β-d-1-thiogalactopyranoside (IPTG), and incubated for an additional 16 h at 20 °C. Recombinant protein was purified by sonicating the harvested cell pellet resuspended in 200 ml of lysis buffer (50 mm Tris-HCl, pH 7.5, 150 mm NaCl, 0.05% Nonidet P-40, and 1 mm DTT), containing 0.1 mg/ml lysozyme, 50 units of DNase I (Thermo Scientific), and 1 tablet of Pierce Protease Inhibitor mixture (Thermo Scientific). After centrifugation at 10,000 rpm for 10 min, the cell supernatant was added to 12.5 ml of glutathione-agarose resin beads (Thermo Scientific) and incubated on ice (4 °C) for 2 h while agitating. The beads were added to a 25-ml Econo-Column® chromatography column (Bio-Rad) and washed with three column volumes of wash buffer (20 mm Tris-HCl, pH 7.5, 150 mm NaCl, and 1 mm DTT). The GST tag was cleaved overnight at 4 °C by addition of PreScission Protease (GE Healthcare), and the ING3 PHD finger was eluted in wash buffer and concentrated to about 3-ml total volume. Protein concentration was determined by absorbance measurement and the *A*_280_ extinction coefficient of ING3_PHD_ (16960 m^−1^ cm^−1^). The purity of the ING3 PHD finger was verified by SDS-polyacrylamide gels stained with GelCode Blue Safe protein stain (Thermo Scientific).

##### Tryptophan Fluorescence Spectroscopy

Tryptophan fluorescence spectra of samples in tryptophan fluorescence buffer (100 mm NaPO_4_, pH 7.5, 150 mm NaCl, and 1 mm DTT) were collected at 25 °C using a Cary Eclipse fluorescence spectrometer (Varian). The samples contained 10 μm ING3 PHD finger protein and progressively increasing concentrations of histone H3 and H4 peptides. The 12- and 14-mer unlabeled histone tail peptides with an amidated C terminus and specific methylation modifications (H3K4me3, H3K4me2, H3K4me1, H3 unmodified, and H4 unmodified) were synthesized by the Peptide Core Facility at the University of Colorado at Denver. After excitation at 295 nm, emission spectra were recorded between 305 and 405 nm with 0.5-nm increments and at a 1-s integration time, averaging over three scans. Each titration experiment was repeated three times, and the average *K_D_* values were calculated using quadratic Equation 1,

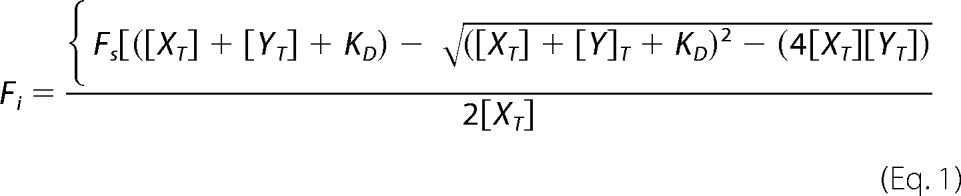
 where *F_i_* is the fluorescence change; *F_s_* is the fluorescence change at saturation of *X_T_* (the total protein concentration); and *Y_T_* is the peptide concentration.

##### Isothermal Titration Calorimetry

ITC measurements were recorded at 5 °C using a MicroCal iTC200 (GE Healthcare) as described previously (55). The wild-type and mutant ING3_PHD_ proteins and histone peptide samples were prepared in a 20 mm NaH_2_PO_4_, pH 7.0, and 150 mm NaCl ITC buffer by dialysis for 24–48 h. Titration experiments were set up for optimal heat of binding reactions by using 100–200 μm ING3_PHD_ in the sample cell, and between 1 and 5 mm histone peptide in the injection syringe. Control experiments were performed under identical conditions to determine the heat of dilution of the titrant peptides into the experimental buffer. This was subtracted from the experimental data as part of data analysis. Data were analyzed using the software ORIGIN 7.0 (OriginLab Corp.). All experiments where binding occurred were performed in triplicate, whereas non-binding experiments were performed in duplicate.

##### HSQC-NMR

Chemical shift perturbation experiments were conducted using 0.5 mm uniformly ^15^N-labeled ING3 PHD finger in buffer containing 20 mm Tris-HCl, pH 6.8, 150 mm NaCl, 10 mm DTT, and 10% D_2_O. Titration mixtures of the ING3_PHD_ protein and each of the modified histone peptides were prepared at concentration ratios of 1:0, 1:0.13, 1:0.26, 1:0.52, 1:1.3, and 1:2.61 in a volume of 35 μl. These mixtures were then transferred into 1.7-mm NMR tubes (Bruker).

Two-dimensional ^15^N HSQC (heteronuclear single quantum coherence) experiments for all samples were run at 25 °C on a 600 MHz Bruker AVANCE III spectrometer equipped with a *z*-gradient 1.7-mm TCI probe at the National Magnetic Resonance Facility at Madison (NMRFAM) using the NMRBot software (57). The NMR data were collected with 1024 × 128 complex data points along the ^1^H and ^15^N dimensions, with acquisition times of 104 and 81 ms, respectively, using eight scans per free induction decay. Normalized chemical shift changes were calculated using Equation 2,

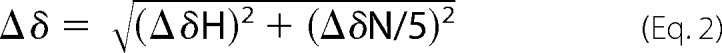
 where ΔδH and ΔδN are the proton and nitrogen change in chemical shift in ppm, respectively.

*K_D_* values were calculated by a nonlinear least squares analysis in KaleidaGraph using Equation 3,

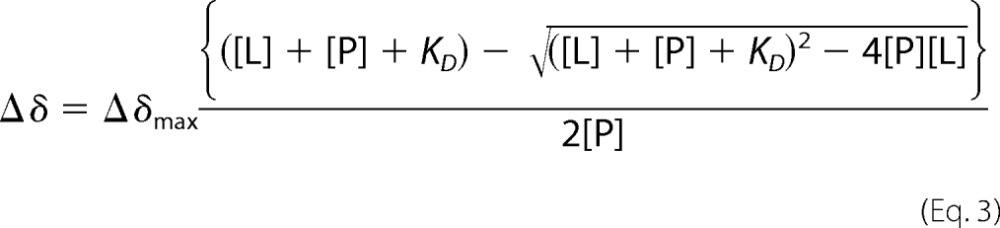
 where [L] is the concentration of the peptide; [P] is the concentration of the protein; Δδ is the observed chemical shift change, and Δδ_max_ is the normalized chemical shift change at saturation.

To obtain the backbone resonance assignments, 1 mm of the ^15^N,^13^C double-labeled ING3 PHD finger was prepared in buffer containing 20 mm Tris-HCl, pH 6.8, 150 mm NaCl, 10 mm DTT, and 10% D_2_O. The Agilent version of ADAPT-NMR (Assignment-directed Data collection Algorithm utilizing a Probabilistic Toolkit in NMR) was used to optimize simultaneous fast data collection and fully automated NMR backbone assignments. In addition to a two-dimensional ^1^H-^15^N HSQC spectrum, ADAPT-NMR recorded six three-dimensional spectra as two-dimensional orthogonal and tilted planes at optimal projection angles as follows: HNCO, HN(CA)CO, HN(CO)CA, HNCA, CBCA(CO)NH, and HN(CA)CB (58). These experiments were collected at 25 °C on a 600 MHz Varian VNMRS spectrometer equipped with a *z*-gradient 5mm cryogenic probe. All two-dimensional planes were processed automatically by ADAPT-NMR with NMRpipe software (59). After less than 1 day, ADAPT-NMR was able to assign all backbone amides (100%). These fully automated assignments were visualized, validated, and further refined by using the ADAPT-NMR Enhancer package (60). This inspection led to the correction of backbone assignments for two residues.

##### MD Simulations

The MD simulations were used to investigate the molecular interactions of the ING3_PHD_ with histone peptide ligands. The published NMR solution structure of the ING3 protein (PDB code 1X4I) was taken from the Protein Data Bank (61). The 1X4I structure was modified with Biopolymer, a structure preparation tool in the SybylX2.1 suite; the N- and the C-terminal residues were capped with *N*-acetyl and *N*-methylamide groups; and protonation types were set for His (ϵ-protonated), Glu (negatively charged), and Lys (positively charged) residues. The Cys and His residues making tetrahedral coordination geometry with structural zinc atoms were treated as special residues in their anionic forms as follows: CY1 residue in zinc-CCCC, CY2, and HIN in zinc-CCCH coordination structures, respectively. The force field parameters for these residues were adopted from ZAFF (62). The side chains of residues Cys-21, Cys-26, Cys-48, and Cys-51 coordinate Zn1 in zinc-CCCC fashion, and Cys-8, Cys-10, Cys-35, and the His-32 coordinate Zn2 in zinc-CCCH fashion; both exhibit tetrahedral geometry.

The starting structures for MD simulations were obtained by flexible protein-peptide docking using the Rosetta FlexPepDock (41, 42) docking program. The docking protocol incorporates iterative cycles of optimization and energy minimization that include full flexibility and rigid body orientation for the peptide backbone, as well as side chain flexibility for both the peptide and the receptor protein. The resulting FlexPepDock complexes were ranked based on the Rosetta full-atom energy function (Rosetta score 12) available within the Rosetta modeling framework (43). The complexes with the highest scores were used as input for the MD simulations.

The FlexPepDock program requires the approximate initial conformation of peptide close to its putative binding pocket. For the H3 peptides, the x-ray crystal structures of ING4 (PDB code 2PNX) and ING5 (PDB code 3C6W), in which the H3K4Me3 peptide is bound to the respective proteins, were used as starting templates. The ING4_PHD_-H3K4me3 and ING5_PHD_-H3K4me3 structures were aligned with the apo-ING3 structures (PDB code 1X4I) based on homology, using the Biopolymer module in Sybyl-X2.1. No structures are available for ING PHD fingers bound to the unmodified histone H4 peptide. Hence, the starting conformation was obtained by docking the unmodified H4 peptide to ING3_PHD_ using the Hex docking program through its webserver (accessed on June 1, 2014) (44). The Hex docking algorithm is based on spherical polar correlations of protein surface shape and electrostatic representations. The initial constraints given to the program were the putative binding residues of the protein observed in our NMR titration studies. The three lowest energy poses of the unmodified histone H4 peptide obtained from the Hex program were fed into the FlexPepDock server to ascertain the binding conformations, and the best pose with the highest Rosetta 12 score was selected for MD simulation, as described above for the H3 peptides.

The MD simulations were performed using the Amber 14 package (63) under isothermal/isobaric (NPT) conditions with Amber ff14SB force field (64) for protein and peptide molecules. The force field parameters for methylated lysine (mono-, di-, and tri-methylated) were obtained by following the standard procedure used in the AMBER force field development utilizing the Mulliken charges calculated as shown previously (55). The backbone torsion parameters are the same as those of the natural lysine residue in the AMBER ff14SB force field.

To prepare the ING3_PHD_ structure for MD simulations, the Leap program from Antechamber tools, AmberTools 14 suite (63, 65), was used to generate the parameter/topology (*prmtop*) and input coordinate (*inpcrd*) files. The net charge of the protein-peptide complexes varied from +3 to +4 depending upon the overall charge of the peptide, and was neutralized by adding Cl^−^ ions at positions of high positive electron potential around the complexes. The system was immersed in a truncated octahedral box of pre-equilibrated TIP3P water molecules (66) in the way that no atoms in the protein-ligand complexes were closer than 16 Å to any of the sides of the water box. The counter ions and solvent molecules were briefly minimized (2500 steps) to remove any bad contacts with the complexes, whereby the protein and peptides were position-restrained using force constant of 100 kcal/(mol·Å^2^), followed by another 2500-step minimization of the whole solvated complex.

To allow the readjustment of solvent molecules to the potential field of the protein-peptide complex, the solvent equilibration step was performed in three stages. The details of this equilibration step have been described previously (55). The production phase with the entire system was carried out under isothermal/isobaric conditions for 10 ns. The SHAKE algorithm (67) was used to constrain bonds involving hydrogen, allowing time steps of 2 fs, for a total of 5,000,000 steps. The trajectory file was written for every 1000 steps (2 ps) resulting in 5000 frames. The cutoff for non-bonded interactions was set to 12 Å in all steps. H-bond interaction analysis was carried out on the 5000 snapshots from the entire 10-ns production phase using the cpptraj program in the AmberTools 14 suite (63, 65), with the cutoff values for distance (3.2 Å) and angle (135°). The pairwise interactions were monitored between the ING3_PHD_-binding site residues (Tyr-8, Gln-13, Val-14, Ser-15, Tyr-16, Gly-17, Glu-18, Met-19, Val-20, Gly-21, Cys-22, Asp-23, Gln-25, Glu-30, and Trp-31) and all histone peptide residues.

To calculate the free energy of binding for each peptide, we used the Molecular Mechanics/Poisson Boltzmann Surface Area (MM/PBSA) method (45), which combines the molecular mechanical energies with the continuum solvent approaches. The molecular mechanical energies represent the internal (bond, angle, and dihedral) energy and van der Waals and electrostatic interactions. The nonpolar contribution to the solvation free energy is determined with solvent-accessible surface area-dependent terms. The method separates nonpolar contribution into two terms as follows: the attractive (dispersion) and repulsive (cavity) interactions. The estimates of vibrational entropies were made using the frequencies from the normal mode analysis with the nmode or NAB module from Amber. The calculations were done using the recently published MMPBSA.py program (46) from Amber Suite.

##### Full-length ING Plasmids

The cDNA of human ING3 was cloned by PCR (Platinum PCR SuperMix High Fidelity (Invitrogen; catalog no. 12532-016) with fwd 5′-ggccAGATCTttgtacctagaagactatctgga-3′ and rev 5′-aggacTCGAGttatttgtgtctgctgcctct-3′ primers) on reverse-transcribed (SuperScript VILO Master Mix; Invitrogen; catalog no. 11755-050) total RNA isolated (TRIzol; Invitrogen; catalog no. 15596-026) from breast carcinoma MCF7 cells. The PCR product was gel-purified, digested with BglII (New England Biolabs) and XhoI (New England Biolabs), and inserted in pCMV 3×FLAG (Stratagene pCMV-3Tag-1A) or pMF retroviral vector (68). The Y362A and W385A H3K4me3-binding defective mutants were generated by site-directed mutagenesis using degenerate primers (Y362A fwd 5′-ccaaatgaacctcgaGCctgcatttgtaatcag-3′ and Y362A rev 5′-ctgattacaaatgcagGCtcgaggttcatttgg-3′; W385A fwd 5′-gattgccctatagaaGCgttccattatggctgc-3′ and W385A rev 5′-gcagccataatggaacGCttctatagggcaatc-3′) and Pfu Turbo (Stratagene). PCR products were digested with DpnI (New England Biolabs) and transformed. All constructs were sequence-verified (Beckman Coulter Genomics).

##### Immunoprecipitation

COS-7 cells were seeded at 500,000 cells per 100-mm dish and transfected with pCMV 3×FLAG-ING3 wild-type (ING3_WT_), Y362A (ING3_Y362A_), or W385A (ING3_W385A_) vectors using Mirus LT1 reagent. Cells were harvested 72 h post-transfection, resuspended in lysis buffer (50 mm Tris-Cl, pH 7.5, 150 mm NaCl, 0.2 mm Na_3_VO_4_, 1% Nonidet P-40, 1 mm PMSF, 1 mm DTT, and protease inhibitors (Roche Applied Science)), incubated with anti-FLAG antibody (M2 clone, Sigma) for 16 h at 4 °C, and immunoprecipitated using Protein G-Sepharose beads (GE Healthcare). Immunoprecipitates were analyzed by immunoblotting with anti-TRRAP (Abcam), anti-TIP60 (SCBT), or anti-FLAG (Sigma) antibodies.

##### Retroviral Expression

HEK293T cells (3 million cells per 100-mm dish) were transfected with 9 μg of indicated pMF constructs (empty, ING3_WT_, ING3_Y362A_, or ING3_W385A_) and 4.5 μg of each VSV-G and gag/pol expressing plasmids using 54 μl of TransIT-LT1 reagent (Mirus). The medium was changed 24 h post-transfection, and supernatants collected at 48 and 72 h post-transfection were filtered (Millipore Millex-HV 0.45-μm PVDF filters) and concentrated (Millipore Amicon Ultra-15).

##### Flow Cytometry Analysis

MCF7 cells were transduced with the indicated retroviral particles in the presence of Polybrene (8 μg/ml). The next day, the medium was refreshed. After 48 h, puromycin selection (1 μg/ml) was applied for 24 h. Cell cycle profiles were assessed as described previously (68). Briefly, MCF7 cells were re-suspended in a propidium iodide staining mixture (0.8% Triton X-100, 50 μg/ml propidium iodide, and 75 μg/ml RNase A) and incubated for 10 min at room temperature. Apoptosis was assessed by staining with annexin V Alexa Fluor488 and propidium iodide (Life Technologies, Inc., V13241). Stained cells were immediately injected into a FACSCalibur; 10,000 cells were analyzed per sample.

## Author Contributions

S.K., S. N., G. C., S. C., M. T., and U. L. M. performed the experiments and together with O. B., C. N. R., J. L. M., S. B., and K. C. G. analyzed the data. K. C. G. and O. B. wrote the manuscript with input from all authors.

## Supplementary Material

Supplemental Data

## Footnotes

7 The abbreviations used are:

PHD: plant homeodomain
ITC: isothermal titration
PDB: Protein Data Bank
r.m.s.d.: root mean square deviation
MD: molecular dynamics
HAT: histone acetyltransferase
ING: INhibitor of Growth
HSQC: heteronuclear single quantum coherence
fwd: forward
rev: reverse.
