# Supplementary material for: Mechanism of Histone H3K4me3 Recognition by the Plant Homeodomain of Inhibitor of Growth 3
Source: J Biol Chem. 2016 Jun 8;291(35):18326–41. doi: 10.1074/jbc.M115.690651 (PMC5000080; doi:10.1074/jbc.M115.690651)
Supplement: Supplemental Data [file supp_291_35_18326__index.html]

Mechanism of Histone H3K4me3 Recognition by the Plant Homeodomain of Inhibitor of Growth 3 — Mechanism of Histone H3K4me3 Recognition by the Plant Homeodomain of Inhibitor of Growth 3 — Histone Recognition by the ING3 PHD Finger — Supplemental Data 

# Mechanism of Histone H3K4me3 Recognition by the Plant Homeodomain of Inhibitor of Growth 3

## Supplemental Data

- Supplemental file 1 (.pdb, 86 KB) - MD simulation of the ING3PHD-H3K4me3 complex
- Supplemental File 1 Description (.doc, 25 KB) - Supplemental File 1 Description
