# Supplementary material for: Mechanism of Histone H3K4me3 Recognition by the Plant Homeodomain of Inhibitor of Growth 3
Source: J Biol Chem. 2016 Jun 8;291(35):18326–41. doi: 10.1074/jbc.M115.690651 (PMC5000080; doi:10.1074/jbc.M115.690651)
Supplement: Supplemental Data [file 10.1074_M115.690651_jbc.M115.690651-2.doc]

**Supplementary File 1**

File name: ING3PHD_H3K4me3_MDsimulation.pdb

Description: MD simulation of the ING3PHD-H3K4me3 complex in PDB format. This time-averaged structure was obtained using 5000 frames of the entire production phase (10 ns) of the MD simulation. The structure was briefly minimized (100 steps) to remove bad contacts using the same conditions as described in Experimental Procedures section ‘Molecular dynamics (MD) simulations’ for the MD simulation.
